# Euthanasia and physician-assisted suicide in patients suffering from psychiatric disorders: a cross-sectional study exploring the experiences of Dutch psychiatrists

**DOI:** 10.1186/s12888-019-2053-3

**Published:** 2019-02-19

**Authors:** Kirsten Evenblij, H. Roeline W. Pasman, Rosalie Pronk, Bregje D. Onwuteaka-Philipsen

**Affiliations:** 1 Department of Public and Occupational Health, VUmc Expertise Center for Palliative Care, Amsterdam Public Health Research Institute, Amsterdam University Medical Centers, Vrije Universiteit, P.O. Box 7057, 1007 Amsterdam, MB Netherlands; 2 Department of General Practice, section Medical Ethics, Amsterdam Public Health Research Institute, Amsterdam University Medical Centers, University of Amsterdam, Amsterdam, The Netherlands

**Keywords:** Assisted suicide, End-of-life care, Epidemiology, Euthanasia, Medical decision making, Psychiatric disorders, Psychiatrists

## Abstract

**Background:**

The medical-ethical dilemmas related to euthanasia and physician-assisted suicide (EAS) in psychiatric patients are highly relevant in an international context. EAS in psychiatric patients appears to become more frequent in the Netherlands. However, little is known about the experiences of psychiatrists with this practice. This study aims to estimate the incidence of EAS (requests) in psychiatric practice in The Netherlands and to describe the characteristics of psychiatric patients requesting EAS, the decision-making process and outcomes of these requests.

**Methods:**

In the context of the third evaluation of the Dutch Euthanasia Act, a cross-sectional study was performed between May and September 2016. A questionnaire was sent to a random sample of 500 Dutch psychiatrists. Of the 425 eligible psychiatrists 49% responded. Frequencies of EAS and EAS requests were estimated. Detailed information was asked about the most recent case in which psychiatrists granted and/or refused an EAS request, if any.

**Results:**

The total number of psychiatric patients explicitly requesting for EAS was estimated to be between 1100 and 1150 for all psychiatrists in a one year period from 2015 to 2016. An estimated 60 to 70 patients received EAS in this period. Nine psychiatrists described a case in which they granted an EAS request from a psychiatric patient. Five of these nine patients had a mood disorder. Three patients had somatic comorbidity. Main reasons to request EAS were ‘depressive feelings’ and ‘suffering without prospect of improvement’. Sixty-six psychiatrists described a case in which they refused an EAS request. 59% of these patients had a personality disorder and 19% had somatic comorbidity. Main reasons to request EAS were ‘depressive feelings’ and ‘desperate situations in several areas of life’. Most requests were refused because the due care criteria were not met.

**Conclusions:**

Although the incidence of EAS in psychiatric patients increased over the past two decades, this practice remains relatively rare. This is probably due to the complexity of assessing the due care criteria in case of psychiatric suffering. Training and support may enable psychiatrists to address this sensitive issue in their work better.

**Electronic supplementary material:**

The online version of this article (10.1186/s12888-019-2053-3) contains supplementary material, which is available to authorized users.

## Background

Euthanasia and/or physician-assisted suicide (EAS) are allowed under strict conditions in five US states, Australia, Colombia, Canada, Luxembourg, Belgium and the Netherlands. Only in the Benelux countries and Canada people who request EAS because of suffering from psychiatric disorders can be eligible for EAS [1–3]. In practice, however, EAS is rarely performed in people with psychiatric disorders. In the Netherlands, 1 % of all 6585 reported EAS cases in 2017 concerned people with psychiatric disorders [4].

The assessment of the statutory due criteria can be complicated when the suffering is caused by a psychiatric disorder [5, 6]. Impaired decisional competence and vulnerability to external pressure (e.g. from relatives) due to the psychiatric illness can obscure the criteria of the ‘voluntary and well-considered nature’ of a request. Moreover, the possibility of spontaneous recovery and the large variety of treatment alternatives make meeting the criteria of ‘no prospect of improvement’ and ‘a lack of reasonable alternatives’ a precarious matter [5, 6].

Although absolute numbers remain small, EAS in psychiatric patients is becoming more frequent in the Netherlands. Between 2008 and 2017, the annual number of EAS cases in psychiatric patients *reported* to the Regional Euthanasia Review Committees increased from 0 to 83 cases [4, 7]. In 2012, the End-of-Life Clinic was founded to provide EAS to patients who meet the statutory due care criteria but whose own physician refuses the request for example because they do not feel competent or feel reluctant to do it themselves even though they do understand or even support the patient’s request [8]. The clinic, which works with mobile teams of qualified physicians and nurses, has become an important stakeholder with regard to EAS in psychiatric practice [9, 10]. In 2017, 62% of the reported psychiatric EAS cases were reported by the End-of-Life Clinic [4].

Until now, little is known about EAS in psychiatric practice. Empirical evidence about patients receiving EAS is concentrated in reviews of EAS cases reported to the Euthanasia Review Committees in the Netherlands and Belgium [11, 12]. Literature on the perspective of psychiatrists on this practice and detailed information about the cases in which a request was refused are lacking, aside from two older Dutch studies reporting on data from 1995 and 2008 [13] (van Helden JJJ E, Rurup ML, van der Heide A, Onwuteaka-Philipsen BD: Physician-assistance in suicide in psychiatric practice in the Netherlands, unpublished) and a study on one psychiatric setting in Belgium [14]. As the public debate, political landscape and professional practice are evolving, up-to-date research on EAS in psychiatric patients is highly relevant. Therefore, this study aims to i) provide estimates of the incidences of EAS requests to psychiatrists and of compliance with such requests and ii) to describe the demographic and clinical characteristics of patients requesting EAS because of psychiatric suffering, the characteristics of the decision making process and outcomes of these requests.

## Methods

### Design and participants

In the context of the third evaluation of the Dutch Euthanasia act, a cross-sectional survey was conducted amongst psychiatrists [15, 16]. A questionnaire was sent to the home or work addresses of a random sample of 500 psychiatrists. Addresses were obtained from a national databank of registered physicians (IMS Health). Inclusion criteria were [1]: working as a psychiatrist in adult patient care for the last year [2], working in the Netherlands and [3] having a registered work or home address.

### Data collection

In May 2016, all selected psychiatrists received a 12-page questionnaire on paper (Additional file 1). The questionnaire was similar to the one used in a nation-wide study amongst Dutch psychiatrists in 1995 [13]. Responding psychiatrists were asked whether they had ever received an EAS request and had ever performed EAS, and how many patients had made an EAS request in the 12 months prior to completing the questionnaire and how many times they performed EAS in the 12 months prior to completing the questionnaire. Detailed information was asked about the most recent case in which they had granted an EAS request and the most recent case in which they had refused an EAS request (if any), including the demographic and clinical characteristics of the patient, reasons for the request (predesignated categories) and characteristics of the decision-making process. Data were collected from May to September 2016. During this time, two reminders were sent.

### Analysis

The survey data were analyzed using IBM SPSS Statistics Software version 22. The number of unique patients requesting EAS and number of unique patients receiving EAS in the previous 12 months as reported by the participating psychiatrists were extrapolated to make an estimation for the number of EAS requests received and performed by all psychiatrists in the Netherlands for a one year period from 2015 to 2016. For this purpose, the number of unique patients requesting EAS (*n* = 91) and number of unique patients receiving EAS (*n* = 7) were multiplied with the weighing factor. The weighing factor (12.40) was calculated by dividing the total number of eligible psychiatrists in the Netherlands (*n* = 2566) by the number of responding psychiatrists (*n* = 207).

## Results

Of the 500 selected psychiatrists, 75 did not meet the selection criteria after all. Of the remaining 425 psychiatrists, 207 responded (response 49%). Some non-responders (29 of the 218) sent a response card providing the reason for not participating: lack of time (*n* = 18), no experience with receiving EAS requests or performing EAS (*n* = 9) and principal objections to EAS (*n* = 2). No psychiatrists reported to have turned down a request due to lack of familiarity with the process or law. Of the 207 responding psychiatrists, 72.8% worked in a private practice, 29.6% in a mental health facility, 11.1% on a psychiatric ward in a general hospital, and 12.3% in another place (more than one answer possible). The mean age was 52 years (range 31–77). Sixty percent was male and 44% indicated that they were religious. The average years of work experience was 17 (range 1–45). Seven psychiatrists received training in palliative care and four were specially trained for the role of independent consultant in the EAS procedure (SCEN-physician).

### Experiences with EAS

Of the 207 responding psychiatrists, 54% had received at least one explicit request for EAS and 4% had performed EAS at least one time throughout his career. Of the psychiatrists who had ever received an explicit request throughout his career, 62% had refused such a request at least one time. Of the psychiatrists 25% had received at least one explicit EAS request in the previous 12 months and 3% of them had actually performed EAS in the previous 12 months. The total number of patients explicitly requesting EAS was estimated to be between 1100 and 1150 for all psychiatrists in the Netherlands in a one year period from 2015 to 2016. An estimated 60 to 70 (approximately 6%) patients received EAS in this period.

### EAS requests that were granted

Of the 112 responding psychiatrists who ever received a request for EAS, nine answered questions on the most recent case in which they granted an explicit EAS request made by a patient with a psychiatric disorder (Table 1). Based on the answers provided by the respondents, it appears that not all psychiatrists who completed questions on the most recent case in which they granted a request, actually performed the EAS themselves.

**Table 1:** The characteristics of patients whose EAS request was granted

	Patient 1	Patient 2	Patient 3	Patient 4	Patient 5	Patient 6	Patient 7	Patient 8	Patient 9
Age	40–50	50–60	50–60	60–70	60–70	60–70	70–80	70–80	80–90
Sex	Female	Male	Male	Female	Male	Female	Female	Male	Male
Main psychiatric diagnosis	Mood disorder	Mood disorder	Personality disorder	Mood disorder	Personality disorder	Personality disorder	Personality disorder	Mood disorder	Mood disorder
Somatic secondary diagnosis	No	No	No	No	No	Yes	Yes	No	Yes
Place of residence	At home	At home	At home	Mental healthcare institution	Mental healthcare institution	At home	At home	Mental healthcare institution	Hospital
Length of time under treatment before first request^a^	1–12 months	> 12 months	> 12 months	< 1 month	1–12 months	< 1 month	> 12 months	< 1 months	1–12 months
Life expectancy	> 12 months	> 12 months	> 12 months	> 12 months	> 12 months	> 12 months	> 12 months	> 12 months	1–5 months
Communication possibilities	Good	Good	Good	Good	Good	Good	Good	Reasonable	Reasonable
Main reasons for the request	Feelings of depression, suffering with no prospect of improvement	Suffering with no prospect of improvement	Feelings of depression, desperate situations in several areas of life, completed life/suffering from life	Feelings of depression, dependence on other people, no longer living independently	Feelings of depression, fear, loneliness, desperate situations in several areas of life, suffering with no prospect of improvement, having no purpose in life	Other physical complaints, physical decline, disability/immobility, loneliness, desperate situations in several areas of life, having no purpose in life,	Physical decline, dependence on other people, loss of or fear of losing control over his or her own life, no longer living independently	Feelings of depression, cognitive decline, loss of or fear of losing control over his or her own life, no longer living independently, suffering with no prospect of improvement, loss of dignity	Pain, fear, suffering with no prospect of improvement, risk of serious complications
Opinion of those close to the patient with regard to the request	Supporting the patient	Supporting the patient	Supporting the patient	Supporting the patient	Supporting the patient	Supporting the patient	Neutral	Supporting the patient	Did not support the request
Patient with decisional competence	Yes	Yes	Yes	Yes	Yes	Yes	Yes	To a certain extent	Yes
Voluntary/well-considered request	Yes	Yes	Yes	Yes	To a certain extent	Yes	Yes	Yes	Yes
Unbearable suffering	Yes	Yes	To a certain extent	Yes	To a certain extent	To a certain extent	Yes	Yes	To a certain extent
Hopeless suffering	Yes	Yes	No	Yes	Yes	To a certain extent	Yes	Yes	Yes
Alternative treatment options	No	No	No	No	To a certain extent	No	No	Yes^b^	No
Other physicians consulted	2 psychiatrists, 1 professor of psychology	3 psychiatrists	No	1 SCEN-physician, 1 psychiatrist	End-of-Life Clinic^d^	1 SCEN-physician, 1 SCEN-physician/psychiatrist, 1 psychiatrist	1 SCEN-physician, 2 psychiatrists	1 SCEN-physician, 1 psychiatrist, 1 clinical geriatrician	1 SCEN-physician, 1 neurosurgeon
Method used^c^	Assisted suicide (performed by general practitioner, or present)	Assisted suicide (performed by End-of-Life Clinic)	Assisted suicide	Euthanasia	Assisted suicide	Assisted suicide (patient referred)	Assisted suicide	Euthanasia	Assisted suicide
Length of decision-making process	–	3 months	8 months	1.5 years	2 months	2 months	1 year	2.5 years	3 months

^a^This question only concerned the time under treatment with the participating psychiatrist. It is very likely that patients have previously been treated by other psychiatrists

^b^At the time of the first request, treatment options were still available. After having tried these treatment options, the patient received EAS 2.5 years after the first request

^c^Euthanasia is defined as death resulting from lethal medication that is administered by a physician with the explicit intention of ending life at the explicit request of the patient. In physician-assisted suicide, the patient self-administers lethal medication that was prescribed by a physician. In the Netherlands both types of EAS are acceptable, in contrast to some other countries with EAS legislation

^d^Although, the responding psychiatrist did not specify the specialty of the End-of-Life Clinic physician, it is likely this concerned a psychiatrist as it is the Clinic’s policy to deploy a psychiatrists in case of a request from a psychiatric patient

#### Characteristics of the patients whose request for EAS was granted

Five of these nine patients were men, four women, their age ranging between 42 to 82 years old. Five had a mood disorder, four a personality disorder. Three patients also had one or more somatic secondary diagnoses. Five patients lived at home.

Three out of nine patients had been treated for at least one year by the responding psychiatrist before their first explicit EAS request, three for at least one month to a year and another three for less than a month. The life-expectancy of all patients at the time of the first explicit EAS request, except for one with a severe and life-limiting comorbidity, was estimated to be more than one year.

#### Main reasons for the EAS request

According to the psychiatrists, the most common reasons for patients to make their EAS request were ‘suffering without prospect of improvement’ and ‘feelings of depression’ (both *n* = 5), ‘desperate situations in several areas of life’ and ‘no longer being able to live independently’ (both *n* = 3). In seven cases, the patient’s relatives supported the request. In one case, relatives adopted a neutral position and in one other case they did not support the patient’s request.

#### Characteristics of the decision-making and practice of EAS

The time taken for the decision-making process from the moment of the first explicit EAS request to its granting, ranged from 2 months to 2.5 years. In all cases the psychiatrists reported that the due care criteria were met, in some cases at least ‘to a certain extent’ (predesignated category). In six cases at least one other psychiatrist was consulted, in five cases (also) a SCEN-physician and in two (also) a medical specialist. In one case no other physician was consulted. After granting the request, seven patients were assisted with suicide, in two cases euthanasia was performed.

### EAS requests that were refused

Of the 112 responding psychiatrists who ever received a request for EAS, 58.9% answered questions on the most recent case in which they refused an explicit EAS request.

#### Characteristics of the patients whose request for EAS was refused

Of the 66 patients whose request was refused, 81.8% were younger than 65 years and 62.5% were female (Table 2). The most common primary psychiatric diagnoses were a personality disorder (59.1%) and a mood disorder (50.0%). Around two-third of the patients had a secondary diagnosis, either psychiatric (45.3%), somatic (9.4%) or both (9.4%). At the time of the first explicit EAS request, the majority (62.1%) lived at home or with relatives. Many (40.9%) patients had been treated for less than one month by the responding physician before they requested EAS. Almost all patients had an estimated life-expectancy of at least 12 months when they made their first explicit EAS request.

**Table 2:** Background characteristics of patients of whom the request for EAS was refused^a^

	*n* = 66	
	%	*n*
Age, years		
16–49 years	42.4	28
50–64	39.4	26
65–79	10.6	7
80+	7.6	5
Gender		
Female	62.5	40
Male	37.5	24
Psychiatric main diagnosis^b^		
Personality disorder	59.1	39
Mood disorder	50.0	33
Psychotic disorder	16.7	11
Autism spectrum disorder	9.1	6
Other	10.6	7
Secondary diagnosis		
No	35.9	23
Yes	64.1	41
Psychiatric	45.3	29
Somatic	9.4	6
Psychiatric and somatic	9.4	6
Place of residence at the time of the request		
Home or with relatives	62.1	41
Mental health facility	24.2	16
Assisted living facility	6.1	4
Psychiatric ward of a general hospital	3.0	2
Other	4.5	3
Time under treatment prior to the first explicit request^c^		
< 1 month	40.9	27
1–12 months	28.8	19
> 12 months	30.3	20
Expected life-expectancy at the time or request		
≤ 12 months	3.0	2
> 12 months	97.0	63
Ability to communicate substantively with the patient		
Good	48.5	31
Reasonably good	28.1	18
Moderately good	20.3	13
Little to none	3.1	2

^a^Missing observations varied between 0 and 2 (0–3%)

^b^More than one answer possible

^c^This question only concerned the time under treatment with the participating psychiatrist. It is very likely that patients have previously been treated by other psychiatrists

#### Main reasons for the EAS request

‘Feelings of depression’ (56.9%) and ‘desperate situations in several areas of life’ (56.9%) were most frequently reported by the responding psychiatrists as the main reasons prompting their patients to make the EAS request. Other frequently reported reasons included ‘suffering without prospect of improvement’ (52.3%), ‘having no purpose in life’ (38.5%) and ‘loneliness’ (27.7%). Some patients requested EAS because of fear in general (16.9%), or fear of losing control over his/her own life (13.8%) or because they did not wish to be a burden for family or relatives (15.4%). In 12.3% of cases, the patient felt he/she had completed or was suffering from his/her life. Complaints of a physical nature such as physical complaints, weakness/fatigue, pain, and disability/immobility were reported in less than 10% of cases.

The patients’ relatives varied in the extend they supported the request. In 28.1% of the cases, they did not support the patient’s request, in 18.8% they did. In another 18.8% the opinions were divided and in 9.4% of the cases relatives adopted a neutral position. In 25.0% of the cases, no relatives were involved.

#### Characteristics of the decision-making and reasons to refuse the EAS request

The duration of the decision-making process before refusing the EAS request ranged from 0 (immediately rejected) to 365 days (Table 3). Of the responding psychiatrists, 40.6% consulted one other physician, 17.2% consulted two other physicians and 6.3% consulted three or more physicians. Yet, 35.9% of the psychiatrists did not consult another physician. In most cases (82.9%) the consulted physician was a psychiatrist. Other physicians consulted were SCEN physicians (12.2%), SCEN physicians who were also a psychiatrists (9.8%), and/or another physician (24.4%).

**Table 3:** Characteristics of the decision making process: duration of decision-making, consultation and reasons to refuse the request^a^

	*n* = 66	
	%	*n*
Duration decision process		
Mean (range)	52 days (0–365)	
Consultation		
Other physician consulted		
No	35.9	23
Yes,	64.1	41
one other physician	40.6	26
two other physicians	17.2	11
three or more other physicians	6.3	4
Specialization of physicians consulted^b^	*n* = 41	
SCEN physician	12.2	5
SCEN physician who is also a psychiatrist	9.8	4
Psychiatrist	82.9	34
Other physician	24.4	10
Reasons to refuse request^c^	*n* = 66	
The due care requirements were not met	75.4	49
Treatment alternatives had not been exhausted	53.1	34
The suffering was not without prospect of improvement	29.7	19
No voluntary and well-considered request	27.7	18
Suffering was not unbearable	10.9	7
Personal objections to EAS in general	23.1	15
Personal objections specific to the case in question	12.3	8
Objections of the family	1.5	1
Other^d^	6.3	4

^a^Missing observations varied between and 2 and 6 (3–9%)

^b^Forty-one psychiatrists consulted another physician and answered this question. More than one answer possible

^c^More than one answer possible, 2 psychiatrists did not provide an explanation for refusing the request. 2 psychiatrists did not specify which due care criteria was not met

^d^Other included: ‘contact with the patient provided the patient with prospect /hope after which the patient withdrew the request’, ‘physical suffering was the main issue’ and ‘I do not assist with suicide but I provide advice and services to enhance the practice of EAS’, ‘No experience with performing EAS’

The most frequently (75.4%) reported reason for refusing the EAS request was that at least one of the due care criteria was not met. In 53.1% of these cases the criteria ‘no reasonable treatment alternatives’ was not met. Almost 70% of these patients could still be treated with psychotropic medication, 55.9% with psychotherapy and 29.4% with electroconvulsive therapy. The criteria ‘suffering without prospect of improvement’ was not met in 29.7% of cases. Moreover, according to the psychiatrists, the request was not ‘voluntary and well-considered’ in 27.7% of cases and the suffering not ‘unbearable’ in another 10.9%.

A substantial percentage (23.1%) of the psychiatrists refused the request because of personal objections to EAS in general (not further specified). Another 12% indicated that they refused the request because of personal objections which mostly concerned the absence of a (good) treatment relationship. Other reasons to refuse a request were ‘objections of the family’ (1.5%) and ‘other reasons’ (6.3%).

#### Outcomes following refusal of EAS request

In 35.4% of the cases the responding psychiatrist referred the patient to another physician after refusing the EAS request: 23.1% of the patients were referred to the End-of-Life Clinic, 10.8% to another physician and 1.5% to an organization which provides information on humane ways of committing suicide (Table 4).

**Table 4:** What happened after the request was refused^a^

	*n* = 66	
	%	*n*
Referred patient after having refused a request?		
No	64.6	42
Yes	35.4	23
to the End of Life Clinic	23.1	15
to another physician	10.8	7
to an organization which provides information on humane ways of committing suicide	1.5	1
Patient personally sought out another doctor who would grant the request after having refusing it^b^		
No	80.5	33
Do not know	12.2	5
Yes	7.3	3
at the End of Life clinic	4.9	2
another physician	2.4	1
Patient died after his or her request was refused		
No	68.8	44
Yes	23.4	15
by suicide	15.6	10
naturally	3.1	2
as a result of euthanasia performed by the End of life clinic	3.1	2
by stopping eating and drinking	1.6	1
Do not know	7.8	5

^a^Missing observations varied between 1 and 2 (1.5–3.0%)

^b^Psychiatrists who answered the first question: ‘Did you refer the patient after having refused his/her request’? with ‘No’ (*n* = 44) were asked if the patient personally sought another doctor (3 missing observations)

The psychiatrists were also asked whether the patient died after his EAS request was refused. In most cases the patient had not died yet at the time of completing the questionnaire (68.8%). In 15.6% of the cases the patient committed suicide, 3.1% of the patients died a natural death, another 3.1% died as a result of EAS performed by the End-of-Life Clinic and 1.6% by stopping to eat and drink.

## Discussion

The total number of psychiatric patients explicitly requesting EAS was estimated to be between 1100 and 1150 for all psychiatrists in a one year period from 2015 to 2016. An estimated 60 to 70 patients received EAS in this period. Nine psychiatrists described a case in which they granted an EAS request from a psychiatric patient. The main reasons to request EAS were ‘suffering without prospect of improvement’ and ‘feelings of depression’. Sixty-six psychiatrists described a case in which they refused an EAS request from a psychiatric patient. The main reasons for those patients to request EAS were ‘feelings of depression’, and ‘desperate situations in several areas of life’. Most requests were refused because the criteria ‘lack of reasonable treatment alternatives’ was not considered to be met.

### Incidence and characteristics of psychiatric patients requesting and receiving EAS over the years

By comparing data from two older Dutch studies with data from the current study, we could identify trends in the incidence of EAS (requests) in the Netherlands. The estimated total number of psychiatric patients explicitly requesting EAS in the Netherlands in a one year period increased from 320 in 1995 to 500 in 2008, to 1100 requests in 2016. The estimated absolute number of patients who received EAS also increased, from 5 in 1995 to 30 in 2008, to 60 in 2016. However, the percentage of EAS requests from psychiatric patients that is granted remains low: 2% in 1995, 6% in 2008 and 5% in 2016 [13].

Although the numbers are small, the characteristics of patients whose request was granted appear to be consistent over time [13]. However, there seems to be a shift in the nature of suffering underlying the request. Whilst in 1995, 10 out of 11 psychiatric patients who received EAS had severe and life-limiting somatic comorbidity, in 2016 only 3 out of 9 psychiatric patients had somatic comorbidity. Due to the verdicts of the euthanasia review committees, a position paper of the Royal Dutch Medical Association and the guideline of the Dutch Association for Psychiatrists, it has become more commonly known that EAS in patients suffering from (solely) psychiatric disorders is legally possible. Psychiatric patients without somatic comorbidity are now more aware that they can request EAS and that their request can be granted. Psychiatrists may also be more willing to perform EAS in patients without somatic comorbidity now that they are aware that these patients can be eligible. Moreover, many cases of EAS in psychiatric patients are performed by the End-of-life clinic which specializes in such complex cases. Another difference is the age of the patients requesting EAS. The number of patients aged 50 years or younger decreased from 7 out of 11 in 1995, to 12 out of 22 in 2008, to only 1 out of 9 in 2016 [13]. The age of patients who received EAS thus increased over the last 20 years.

### Granted vs refused EAS requests

Overall, there were no substantial differences between characteristics of patients whose EAS request was refused and those whose request was granted in 2016. Though the limited number of granted cases calls for prudence, the prevalence of somatic comorbidity amongst patients whose EAS requests was granted, appeared slightly higher compared to those whose request was refused. A study by Kim et al. reported a somewhat higher prevalence of somatic comorbidity amongst psychiatric patients who received EAS (60%) compared to our findings [12]. However, in that study some of the EAS cases were carried out by a general practitioner and general practitioners are more aware of somatic comorbidity. Moreover, psychiatric patients requesting their general practitioner for EAS instead of their psychiatrist possibly suffer less severely from psychiatric and more from somatic disorders. Also, a different data collection method was used. We asked psychiatrists whether there were any *important* somatic secondary diagnoses while Kim et al. reviewed psychiatric EAS cases that were reported to the Regional Euthanasia Review Committees by physicians. Physicians tend to report very comprehensively on the nature of suffering, including any somatic comorbidity.

Although the Dutch Euthanasia law does not differentiate between somatic or psychiatric suffering, it appears that physicians do. In 2015, a study among Dutch physicians showed that physicians considered it significantly less likely to assist in the EAS of a patient suffering from a psychiatric disease than of a terminally ill patient with a severe somatic disease [17]. This could be attributed to the complexity of meeting the statutory due care criteria for these patients. Indeed, our study revealed that the main reason psychiatrists provided for refusing an EAS request was that the due care criteria were not met. Given the large variety of treatment options for psychiatric disorders, deciding that there are ‘no reasonable alternatives’ can be a difficult and subjective process depending on what the psychiatrist considers reasonable and effective treatment [5]. Moreover, the unpredictability of the development of psychiatric disorders and the possibility of long-term remission, make it difficult to judge whether the suffering is ‘without prospect of improvement’[18–20]. Furthermore, suicidal ideation can be an expression of the psychiatric disorder and may fluctuate over time [21]. Also, some psychiatrists argue that people with psychiatric disorders are by definition incompetent to make decisions about EAS, although others do not share this opinion [22]. Given these medical and ethical issues, it is possible that psychiatrists assume that psychiatric patients cannot meet the due care criteria. Consequently, some psychiatric patients with an EAS request turn to the End-of-Life Clinic, where for that matter, only 10% of the requests from psychiatric patients is eventually granted [10]. This low percentage shows once again how difficult it is for the due care criteria to be met in psychiatric patients requesting EAS.

### Strengths and limitations

This study provides up-to-date information about the evolving practice of EAS in patients with psychiatric disorders from the perspective of practicing psychiatrists. The strengths of this study are the use of a nationwide random sample of psychiatrists and the re-use of an existing questionnaire which allowed us to put our results into an historic perspective. Another strength is that we are the first to report separately on the characteristics of cases in which the EAS request was refused. Although this study describes the Dutch situation, the medical and ethical dilemmas are highly relevant in an international context.

When interpreting the results of this study, it is important to realize that data were gathered from psychiatrists only. Practice indicates, however, that other physicians (e.g. general practitioners), also receive EAS requests from psychiatric patients. This may have led to an underestimation of the number of requests and the frequency of compliance with these requests in psychiatric patients, although there is no literature available on requests from psychiatric patients outside psychiatric practice to support this. Furthermore, it is possible that the characteristics of the psychiatric patients requesting psychiatrists for EAS are not generalizable to those who applied their request to other physicians. In addition, it is important to note that granting an EAS request is not tantamount to actually performing EAS. This led to a discrepancy in the number of psychiatrists who filled out questions on a granted cases and the number psychiatrists reporting to have ever performed EAS. A limitation of this study is the retrospective nature of the questionnaire which may have led to recall bias. Another limitation is the rather low response rate and the limited number of cases described. This may have led to selection bias. However, the numbers of EAS in psychiatric practice are low and our findings are largely consistent with existing evidence [10–14]. Therefore, it is less likely that the low response rate has compromised the validity of our findings.

## Conclusions

Although the incidence of EAS (requests) from psychiatric patients increased over the past two decades, EAS in psychiatric patients remains relatively rare. This is most likely due to difficulties for psychiatric patients to meet the due care criteria and for psychiatrists to determine whether the criteria are met. Training and support might enable psychiatrists to address this complex and sensitive issue in their work better.

Box 1 Statutory due care criteria [1] Under section 2 (1) of the Act, physicians who carry out an EAS request must:

- be satisfied that the patient’s request is voluntary and well-considered;
- be satisfied that the patient’s suffering is unbearable, with no prospect of improvement;
- have informed the patient about his situation and prognosis;
- have come to the conclusion, together with the patient, that there is no reasonable alternative in the patient’s situation;
- have consulted at least one other, independent physician, who must see the patient and give a written opinion on whether the due care criteria set out in (a) to (d) have been fulfilled;
- have exercised due medical care and attention in terminating the patient’s life or in assisting in his suicide.

## Additional file

Additional file 1: Questionnaire. English translation of the questionnaire used in the cross-sectional survey study. (DOCX 300 kb)

## Abbreviation

EAS: Euthanasia and physician-assisted suicide
