# Supplementary material for: Euthanasia and physician-assisted suicide in patients suffering from psychiatric disorders: a cross-sectional study exploring the experiences of Dutch psychiatrists
Source: BMC Psychiatry. 2019 Feb 19;19:74. doi: 10.1186/s12888-019-2053-3 (PMC6381744; doi:10.1186/s12888-019-2053-3)
Supplement: Supplementary file 1 — Questionnaire. English translation of the questionnaire used in the cross-sectional survey study. (DOCX 300 kb) [file 12888_2019_2053_MOESM1_ESM.docx]

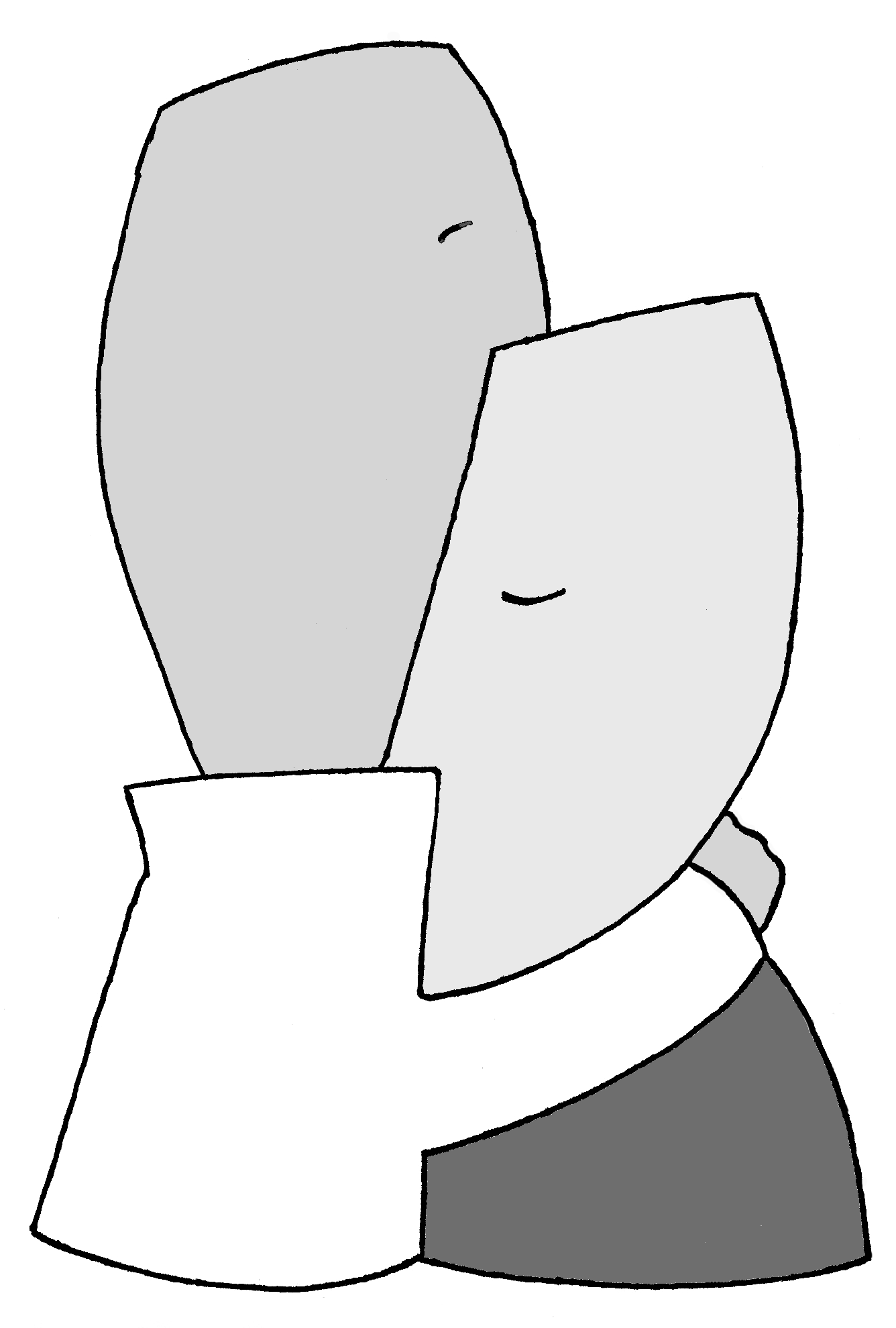
Evaluation Termination of life on request
and assisted suicide act.

Questionnaire for psychiatrists


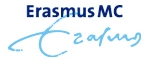

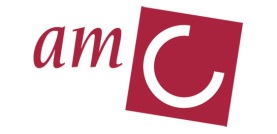

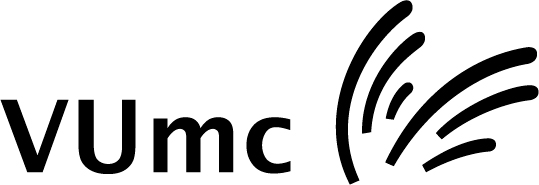

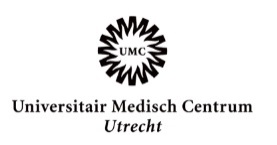


**Instruction**

**You will remain completely anonymous when completing this questionnaire.**

With most questions you only have to tick **one** box. If a question allows you to tick more than one, this will be clearly stated.

If you are asked for your experiences in the past year, we mean the last **12 months** prior to completing this questionnaire.

It is important that you make use of the following definitions when answering the questions:

**Assisted suicide**: the patient terminates his or her own life by ingesting lethal drugs, which are provided by a physician at the patient’s explicit request.

**Euthanasia**: the termination of the life of a patient by a physician through the administration of lethal drugs at the patient his or her explicit request.

**Attention:** For the sake of brevity, in the questionnaire, we use only the term assisted suicide. However, this refers to both euthanasia and assisted suicide.

If you have any questions or comments in response to this study or this questionnaire, please contact:

**K. Evenblij, MSc (e-mail: k.evenblij@vumc.nl)**

**Prof.dr. B.D. Onwuteaka-Philipsen (e-mail: b.philipsen@vumc.nl)**

| A. Starting | | | | |  |
| --- | --- | --- | --- | --- | --- |
|  | Have you been working as a psychiatrist in the past year? | | - Yes | |  |
|  |  |  | - No 🡪 you do not have to complete this questionnaire. Please do return the questionnaire using the response envelope | |  |
|  | 1. How long have you been working as a psychiatrist? | | year | |  |
|  | 1. How many hours do you work as a psychiatrist? | | hours a week | |  |
|  | 1. Where do you work as a psychiatrist?   *(One or more answers possible)* | | - Private practice | |  |
|  |  |  | - Mental health facility | |  |
|  |  |  | - Psychiatric ward in general hospital | |  |
|  |  |  | - Another place: | |  |
|  | Are you working as a palliative care consultant or SCEN-physician?  *(One or more answers possible)* | | - No | |  |
|  |  |  | - Yes, SCEN-physician | |  |
|  |  |  | - Yes, palliative care consultant | |  |
|  |  |  | - Yes, part of a palliative care team | |  |
| B. Experiences with assisted suicide | | | | | |
|  | 1. Did it occur that a patient requested you for assisted suicide in due time (at which the patient did not want it in the near future but for example when the suffering becomes unbearable)? | | - Yes | | |
|  |  |  | - No 🡪 Continue with **question 2** | | |
|  | 1. **If yes,** in the past year, how many patients have request you for assisted suicide in due time? | | patients | | |
|  | 1. Did it occur that a patient made an explicit request for assisted suicide in the foreseeable future? | | - Yes | | |
|  |  |  | - No 🡪 Continue with part C | | |
|  | 1. Have you ever assisted in the suicide of a patient (at the patient’s explicit request)? | | - Yes | | |
|  |  |  | - No | | |
|  | 1. In the past year, how many of your patients have made an explicit request for assisted suicide in the foreseeable future? | | patients | | |
|  |  |  | - None 🡪 Continue with part C | | |
|  | 1. In the past year, how many patients did you assist in suicide? | | patients | | |
|  | 1. What was the primary cause of suffering underlying the explicit requests received in the past year (question 3a and 3b)? *(If a patient fits in more than one category, chose the most applicable option)* | | Number of requests received | Number of requests granted | |
|  | **Suffering from**: | Only a psychiatric cause |  |  | |
|  |  | A psychiatric and somatic cause |  |  | |
|  |  | Only a somatic cause |  |  | |
|  |  | There was no psychiatric or somatic cause for the suffering |  |  | |

| C. Last request for assisted suicide that you granted | | | | | | | | | | | | | |
| --- | --- | --- | --- | --- | --- | --- | --- | --- | --- | --- | --- | --- | --- |
| The following questions concern the last time that you assisted in the suicide of a patient with a psychiatric disorder at his or her explicit request. In case you never granted such a request, please indicate this below and continue with part D.   - Not applicable, I have never granted such a request. | | | | | | | | | | | | | |
|  | What was the age of the patient at the time of carrying out the request? | | year | | | | | | | | | | |
|  | What was the sex of the patient? | | - Male | | | | | - Female | | | | |  |
|  | The psychiatric main diagnosis was  *(One or more answers possible)* | | - A mood disorder | | | | | | | | | | |
|  |  |  | - A psychotic disorder | | | | | | | | | | |
|  |  |  | - A personality disorder - Other: | | | | | | | | | | |
|  | Where there important secondary diagnoses?  *(One or more answers possible)* | | - No | | | | | | | | | | |
|  |  |  | - Yes, a psychiatric secondary diagnosis: | | | | | | | | | | |
|  |  |  | - Yes, a somatic secondary diagnosis: | | | | | | | | | | |
|  |  |  |  | | | | | | | | | | |
|  | At the time of the request, the patient was staying at | | - Home or with relatives | | | | | | | | | | |
|  |  |  | - A mental health facility | | | | | | | | | | |
|  |  |  | - A psychiatric ward of a general hospital | | | | | | | | | | |
|  |  |  | - A somatic ward of a general hospital | | | | | | | | | | |
|  |  |  | - A hospice | | | | | | | | | | |
|  |  |  | - Other: | | | | | | | | | | |
|  | How long was the patient under treatment prior to his/her first explicit request for assisted suicide in the foreseeable future? | | - Less than one month | | | | | | | | | | |
|  |  |  | - 1 to 12 months | | | | | | | | | | |
|  |  |  | - More than 12 months | | | | | | | | | | |
|  | How did you estimate the life expectancy of the patient at the time of the request? | | - Less than 1 week | | | | | | | - 6 to 12 months | | | |
|  |  |  | - 1 to 4 weeks | | | | | | | - More than 12 months | | | |
|  |  |  | - 1 to 5 months | | | | | | | |  | | |
|  | To what extent was substantive communication with the patient possible at the time of decision making? | | - Good | | | | | | | - Moderately good | | | |
|  |  |  | - Reasonably good | | | | | | | - Little to none | | | |
|  | What were the main reasons for the patient to make the request for assisted suicide?  *(One or more answers possible)* | | | | | | | | | | | | |
|  | - General weakness/fatigue | | | | - Dependence on medication | | | | | | | | |
|  | - Shortness of breath | | | | - Dependence on people | | | | | | | | |
|  | - Pain | | | | - Loss of or fear of losing control over his or her own life | | | | | | | | |
|  | - Other physical complaints | | | | - Not wishing to be a burden for his or her family or those around him or her | | | | | | | | |
|  | - Depressive feelings | | | | - Not being able to live independently | | | | | | | | |
|  | - Fear | | | | - Suffering without prospect of improvement | | | | | | | | |
|  | - Cognitive decline | | | | - Having no goal in life | | | | | | | | |
|  | - Physical decline | | | | - Loss of dignity | | | | | | | | |
|  | - Disability/immobility | | | | - Completed life/suffering from life | | | | | | | | |
|  | - Death of someone close to him/her | | | | - Other: | | | | | | | | |
|  | - Loneliness | | | |  | | | | | | | | |
|  | - Ending up in a rut in several areas of life (e.g.: work/financial/relationship) | | | |  |  |  |  |  |  |  |  |  |
|  | Did it concern current suffering or fear of suffering in the future? | | | | | - Current suffering | | | | | | | |
|  |  |  |  |  |  | - Fear of future suffering | | | | | | | |
|  | When the explicit request for assisted suicide in the foreseeable future was made for the first time, were there any psychiatric treatment alternatives which the patient declined?  *(One or more answers possible)* | | | | | - No | | | | | | | |
|  |  |  |  |  |  | - Yes, psychotropic medication | | | | | | | |
| \ |  |  |  |  |  | - Yes, psychotherapy | | | | | | | |
|  |  |  |  |  |  | - Yes, electroconvulsive therapy | | | | | | | |
|  |  |  |  |  |  | - Yes, other: | | | | | | | |
|  | | Was there, at your discretion, at the time of this request | | | | | No | | Somewhat | | | Yes | |
|  | | 1. A patient with decisional competence? | | | | |  | |  | | |  | |
|  | | 1. Unbearable suffering? | | | | |  | |  | | |  | |
|  | | 1. Hopeless suffering? | | | | |  | |  | | |  | |
|  | | 1. A voluntary and well-considered request? | | | | |  | |  | | |  | |
|  | | 1. Alternative treatment options? | | | | |  | |  | | |  | |
|  | | Did you consult one or more other physicians in response to the request for assisted suicide of this patient? | | - Yes, one other physician | | | | | | | | | |
|  | |  |  | - Yes, more than one other physician | | | | | | | | | |
|  | |  |  | - No 🡪 Continue with **question 15** | | | | | | | | | |
|  | | How many other physicians did you consult?  *(One or more answers possible)* | | SCEN-physician(s) | | | | | | | | | |
|  | |  |  | SCEN-physician(s) who is also a psychiatrist | | | | | | | | | |
|  | |  |  | Psychiatrist(s) | | | | | | | | | |
|  | |  |  | Other physician(s): | | | | | | | | | |
|  | |  |  |  | | | | | | | | | |
|  | | What was the opinion of relatives with regard to the request? | | - Not applicable, there were no relatives involved | | | | | | | | | |
|  | |  |  | - They adopted a neutral position | | | | | | | | | |
|  | |  |  | - They supported the patient in the request | | | | | | | | | |
|  | |  |  | - They did not support the patient’s request | | | | | | | | | |
|  | |  |  | - The opinions were divided | | | | | | | | | |
|  | | 1. Did you assisted in suicide or did you perform euthanasia? | | - Assisted in suicide | | | | | | | | | |
|  | |  |  | - Performed euthanasia | | | | | | | | | |
|  | | 1. Were you present during the assisted suicide? | | - Yes | | | | | | | | | |
|  | |  |  | - No | | | | | | | | | |
|  | How long did the decision-making process take from the first explicit request until the assisted suicide. | | | days/weeks/months  *(strike out what does not apply)* | | | | | | | | | |

| D. Last request for assisted suicide that you refused | | | | | | | | | | | | | | | |
| --- | --- | --- | --- | --- | --- | --- | --- | --- | --- | --- | --- | --- | --- | --- | --- |
| The following question concern the last time that you refused an explicit request for assisted suicide from a patient with a psychiatric disorder. In case you never refused such a request, please indicate below and continue with part E.   - Not applicable, I have never refused such a request. | | | | | | | | | | | | | | | |
|  | What was the age of the patient at the time of carrying out the request? | | year | | | | | | | | | | | |  |
|  | What was the sex of the patient? | | - Male | | | | | | - Female | | | | |  |  |
|  | The psychiatric main diagnosis was  *(One or more answers possible)* | | - A mood disorder | | | | | | | | | | | |  |
|  |  |  | - A psychotic disorder | | | | | | | | | | | |  |
|  |  |  | - A personality disorder - Other: | | | | | | | | | | | |  |
|  | Where there important secondary diagnoses?  *(One or more answers possible)* | | - No | | | | | | | | | | | |  |
|  |  |  | - Yes, a psychiatric secondary diagnosis: | | | | | | | | | | | |  |
|  |  |  | - Yes, a somatic secondary diagnosis: | | | | | | | | | | | |  |
|  |  |  |  | | | | | | | | | | | |  |
|  | At the time of the request, the patient was staying at | | - Home or with relatives | | | | | | | | | | | |  |
|  |  |  | - A mental health facility | | | | | | | | | | | |  |
|  |  |  | - A psychiatric ward of a general hospital | | | | | | | | | | | |  |
|  |  |  | - A somatic ward of a general hospital | | | | | | | | | | | |  |
|  |  |  | - A hospice | | | | | | | | | | | |  |
|  |  |  | - Other: | | | | | | | | | | | |  |
|  | How long was the patient under treatment prior to his/her first explicit request for assisted suicide in the foreseeable future? | | - Less than one month | | | | | | | | | | | |  |
|  |  |  | - 1 to 12 months | | | | | | | | | | | |  |
|  |  |  | - More than 12 months | | | | | | | | | | | |  |
|  | How did you estimate the life expectancy of the patient at the time of the request? | | - Less than 1 week | | | | | | | | - 6 to 12 months | | | |  |
|  |  |  | - 1 to 4 weeks | | | | | | | | - More than 12 months | | | |  |
|  |  |  | - 1 to 5 months | | | | | | | | |  | | |  |
|  | To what extent was substantive communication with the patient possible at the time of decision making? | | - Good | | | | | | | | - Moderately good | | | |  |
|  |  |  | - Reasonably good | | | | | | | | - Little to none | | | |  |
|  | What were the main reasons for the patient to make the request for assisted suicide?  *(One or more answers possible)* | | | | | | | | | | | | | |  |
|  | - General weakness/fatigue | | | | - Dependence on medication | | | | | | | | | |  |
|  | - Shortness of breath | | | | - Dependence on people | | | | | | | | | |  |
|  | - Pain | | | | - Loss of or fear of losing control over his or her own life | | | | | | | | | |  |
|  | - Other physical complaints | | | | - Not wishing to be a burden for his or her family or those around him or her | | | | | | | | | |  |
|  | - Depressive feelings | | | | - Not being able to live independently | | | | | | | | | |  |
|  | - Fear | | | | - Suffering without prospect of improvement | | | | | | | | | |  |
|  | - Cognitive decline | | | | - Having no goal in life | | | | | | | | | |  |
|  | - Physical decline | | | | - Loss of dignity | | | | | | | | | |  |
|  | - Disability/immobility | | | | - Completed life/suffering from life | | | | | | | | | |  |
|  | - Death of someone close to him/her | | | | - Other: | | | | | | | | | |  |
|  | - Loneliness | | | |  | | | | | | | | | |  |
|  | - Ending up in a rut in several areas of life (e.g.: work/financial/relationship) | | | |  |  |  |  |  |  |  |  |  |  |  |
|  |  | | | |  | | | | | | | | | |  |
|  | Did it concern current suffering or fear of suffering in the future? | | | | | - Current suffering | | | | | | | | |  |
|  |  |  |  |  |  | - Fear of future suffering | | | | | | | | |  |
|  | Were there, when the explicit request for assisted suicide in the foreseeable future was made for the first time, any psychiatric treatment alternatives that were declined by the patient?  *(One or more answers possible)* | | | | | - No | | | | | | | | |  |
|  |  |  |  |  |  | - Yes, psychotropic medication | | | | | | | | |  |
| \ |  |  |  |  |  | - Yes, psychotherapy | | | | | | | | |  |
|  |  |  |  |  |  | - Yes, electroconvulsive therapy | | | | | | | | |  |
|  |  |  |  |  |  | - Yes, other: | | | | | | | | |  |
|  | | Was there, at your discretion, at the time of this request | | | | | No | | | Somewhat | | | Yes | |  |
|  | | 1. A patient with decisional competence? | | | | |  | | |  | | |  | |  |
|  | | 1. Unbearable suffering? | | | | |  | | |  | | |  | |  |
|  | | 1. Hopeless suffering? | | | | |  | | |  | | |  | |  |
|  | | 1. A voluntary and well-considered request? | | | | |  | | |  | | |  | |  |
|  | | 1. Alternative treatment options? | | | | |  | | |  | | |  | |  |
|  | | Did you consult one or more other physicians in response to the request for assisted suicide of this patient? | | - Yes, one other physician | | | | | | | | | | |  |
|  | |  |  | - Yes, more than one other physician | | | | | | | | | | |  |
|  | |  |  | - No 🡪 Continue with **question 15** | | | | | | | | | | |  |
|  | | How many other physicians did you consult?  *(One or more answers possible)* | | SCEN-physician(s) | | | | | | | | | | |  |
|  | |  |  | SCEN-physician(s) who is also a psychiatrist | | | | | | | | | | |  |
|  | |  |  | psychiatrist(s) | | | | | | | | | | |  |
|  | |  |  | other physician(s): | | | | | | | | | | |  |
|  | |  |  |  | | | | | | | | | | |  |
|  | | What was the opinion of relatives with regard to the request? | | - Not applicable, there were no relatives involved | | | | | | | | | | |  |
|  | |  |  | - They adopted a neutral position | | | | | | | | | | |  |
|  | |  |  | - They supported the patient in the request | | | | | | | | | | |  |
|  | |  |  | - They did not support the patient’s request | | | | | | | | | | |  |
|  | |  |  | - The opinions were divided | | | | | | | | | | |  |
|  | What was the reason for refusing the request? | | | - Personal objections to assisted suicide in general | | | | | | | | | | |  |
|  |  |  |  | - Objections of the family | | | | | | | | | | |  |
|  |  |  |  | - The due care criteria were not met, namely**:** | | | | | | | | | | |  |
|  |  |  |  | - Personal objections specific to the case in question**:** | | | | | | | | | | |  |
|  |  |  |  | - Other: | | | | | | | | | | |  |
|  | 1. Did you refer the patient after you had refused his or her request for assisted suicide? | | | - Yes, to the End-of-Life clinic | | | | | | | | | | |  |
|  |  |  |  | - Yes, to another physician | | | | | | | | | | |  |
|  |  |  |  | - Yes, to an organization which provides information on humane ways of committing suicide | | | | | | | | | | |  |
|  |  |  |  | - No | | | | | | | | | | |  |
|  | 1. **If not,** Did the patient him or herself sought out another physician who would grant the request? | | | - Yes, to the End-of-Life clinic | | | | | | | | | | |  |
|  |  |  |  | - Yes, to another physician | | | | | | | | | | |  |
|  |  |  |  | - Do not know | | | | | | | | | | |  |
|  |  |  |  | - No | | | | | | | | | | |  |
|  | 1. **If not**, what do you think was the reason that the patient did not seek out another physician?? | | | | | | | | | | | | | |  |
|  |  | | | | | | | | | | | | | |  |
|  | How long did the decision-making process take from the first explicit request until the assisted suicide. | | | days/weeks/months  *(strike out what does not apply)* | | | | | | | | | | |  |
| 1. ~~\~~ | Did the patient die after his or her request was refused? | | | - No 🡪 Continue with part **E** | | | | | | | | | | |  |
|  |  |  |  | - Do not know 🡪 Continue with part **E** | | | | | | | | | | |  |
|  |  |  |  | - Yes; | | | | - A natural death | | | | | | |  |
|  |  |  |  |  | | | | - As a result of assistance in suicide by the End-of-Life clinic | | | | | | |  |
|  |  |  |  |  | | | | - As a result of assistance in suicide by another physician | | | | | | |  |
|  |  |  |  |  | | | | - By stopping eating and drinking | | | | | | |  |
|  |  |  |  |  | | | | - By suicide | | | | | | |  |

| E. Closing | | | | |
| --- | --- | --- | --- | --- |
|  | 1. What is your sex and your age? | Sex: | - Male | - Female |
|  |  | Age: | year | |
|  | 1. Are you religious? | - No | | |
|  | **If yes,** what religion? | - Yes: | | |
|  | Have you had any extra training (apart from your regular education) in the field of palliative care? | - No | | |
|  |  | - Yes, “kaderopleiding palliatieve zorg” | | |
|  |  | - Yes, other: | | |

**Finally**Room for comments:

**Thank you very much for completing this questionnaire.**
